# Preparation and Photocatalytic Activity of Potassium-Incorporated Titanium Oxide Nanostructures Produced by the Wet Corrosion Process Using Various Titanium Alloys

**DOI:** 10.3390/nano5031397

**Published:** 2015-08-21

**Authors:** So Yoon Lee, Choong Hyun Lee, Do Yun Kim, Jean-Pierre Locquet, Jin Won Seo

**Affiliations:** 1 Department of Materials Engineering, KU Leuven, Kasteelpark Arenberg 44—Bus 2450, Leuven B-3001, Belgium; E-Mail: soyoon.lee@mtm.kuleuven.be; 2 Department of Materials Engineering, the University of Tokyo, 7-3-1 Hongo, Tokyo 113-8656, Japan; E-Mail: clee@us.ibm.com; 3 Laboratory of Photovoltaic Materials and Device, Department of Electrical Sustainable Energy, Delft University of Technology, Delft 2628CD, The Netherlands; E-Mail: d.kim@fz-juelich.de; 4 Laboratory of Solid-State Physics and Magnetism, KU Leuven, Celestijnenlaan 200D, Leuven B-3001, Belgium; E-Mail: jeanpierre.locquet@fys.kuleuven.be

**Keywords:** titanium, Ti–Al–V, TiNi (TN), nanostructures, wet corrosion process (WCP), photocatalysis

## Abstract

Nanostructured potassium-incorporated Ti-based oxides have attracted much attention because the incorporated potassium can influence their structural and physico-chemical properties. With the aim of tuning the structural and physical properties, we have demonstrated the wet corrosion process (WCP) as a simple method for nanostructure fabrication using various Ti-based materials, namely Ti–6Al–4V alloy (TAV), Ti–Ni (TN) alloy and pure Ti, which have 90%, 50% and 100% initial Ti content, respectively. We have systematically investigated the relationship between the Ti content in the initial metal and the precise condition of WCP to control the structural and physical properties of the resulting nanostructures. The WCP treatment involved various concentrations of KOH solutions. The precise conditions for producing K-incorporated nanostructured titanium oxide films (nTOFs) were strongly dependent on the Ti content of the initial metal. Ti and TAV yielded one-dimensional nanowires of K-incorporated nTOFs after treatment with 10 mol/L-KOH solution, whereas TN required a higher concentration (20 mol/L-KOH solution) to produce comparable nanostructures. The obtained nanostructures revealed a blue-shift in UV absorption spectra due to the quantum confinement effects. A significant enhancement of the photocatalytic activity was observed via the chromomeric change and the intermediate formation of methylene blue molecules under UV irradiation. This study demonstrates the WCP as a simple, versatile and scalable method for the production of nanostructured K-incorporated nTOFs to be used as high-performance photocatalysts for environmental and energy applications.

## 1. Introduction

For several decades, nanostructured metal oxides have been one of the most extensively studied materials because of their unique and diverse physico-chemical properties and their potential for diverse applications [1,2,3,4,5,6,7,8]. In particular, nanostructured Ti and Ti-based alloy oxides have widely been researched for applications such as photovoltaic cells, batteries, photochromic and electrochromic devices, highly efficient catalysts, gas sensors, biomaterials, biosensors and pigments, owing to their semiconducting properties, high dielectrical permittivity, ion-exchange ability, high refractive index, and superior biocompatibility [9,10,11,12,13,14,15,16,17].

In particular, nanostructured alkali metal incorporated titanates containing A–Ti–O (A = alkali element and/or H) bonds have been produced as nanotubes, nanorods, nanofibers, and nanosheets. They have attracted considerable interest because of their unique layered structure [18,19,20,21,22]. Since the presence of the alkali metal in these titanates can significantly influence the physical properties, they have so far been studied extensively in order to tune their physical properties beyond the limitations of conventional materials, as reported for instance in applications like electrical devices, photocatalyst, energy storage, gas sensors, and photovoltaics [23,24,25,26]. Among them, potassium (K)-incorporated titanates have been of particular interest due to their specific photochemical properties or their artificial cage type structure [27,28,29,30]. Although these K-incorporated nanostructured titanium oxides films (nTOFs) have been one of the leaders in this new class of materials, their synthesis has still limitations to tackle. Although several synthesis methods such as the sol-gel process, template-mediated process, and hydrothermal route have been developed to obtain the desired structural, chemical and physical properties, nanostructure fabrication has generally involved complicated processes, low reproducibility and high costs for well-controlled chemical modification [21,22,23,24,25,26,27,28,29,30,31,32,33,34]. Since the physical properties can be greatly affected by the size and morphology of the nanostructures as well as the precise incorporated K amount, several processes had to be combined, exacerbating these drawbacks. Despite great expectations about these materials, there is still a lack of systematic research and strategy for nanostructure fabrication while simultaneously controlling the physical properties. This bottleneck strongly limits their fast implementation in potential applications [35,36,37]. Hence, elaborating a process that allows for easy synthesis and a simple tuning of the desired morphology and properties via a single-step process is currently one of the key issues in this research field.

As previously reported, K-incorporated nTOFs could be produced via the wet corrosion process (WCP) by using pure Ti metal [23,38,39]. WCP is a simple one-step process that uses alkali solutions. Moreover, this process produces nanostructures as a thin film onto a metal surface directly, which is particularly advantageous for diverse applications. Since the nanostructures are immobilized on the metal surface, the problem of homogenous dispersion, as very often occurs with powders, can be avoided. The previous works on WCP demonstrated that it could control the morphology as well as improve the electrical properties of K-incorporated nTOFs by using various concentrations of the KOH aqueous solutions without any additional processes. Moreover, a direct correlation between structural, and physical properties and the incorporated K amount was illustrated. In the present work, we extended our studies to different Ti-based metal substrates and, for the first time, systematically investigated the characteristics of the resulting nTOFs depending on the initial Ti content prior to the WCP. The results clearly indicate direct dependence of the nanostructure fabrication on the Ti content in the initial material. Moreover, the optical property of the nTOFs could be correlated with the structural properties. Understanding the relationship between the Ti content of the initial metal and the condition of WCP to control the structural and physical properties makes it possible to produce nTOFs on demand, for various applications.

Recently, Ti-based materials have attracted significant attention as multifunctional semiconductor photocatalysts for eco-friendly alternative technology, in particular because of their chemical and biological inertness, photostability and low cost of production [40,41]. So far, a number of effective approaches have been developed to enhance the photocatalytic activity by tuning the structural, chemical and physical properties of the modified Ti-based materials [42,43,44]. However, the relationship between the effect of nanostructure fabrication and the resulting photocatalytic property is still unclear. In this study we demonstrate that the WCP yields highly reproducible nanostructures of K-incorporated nTOFs with high potential for treating water containing organics, removing metals from water and splitting water.

## 2. Results and Discussion

**Figure 1:**
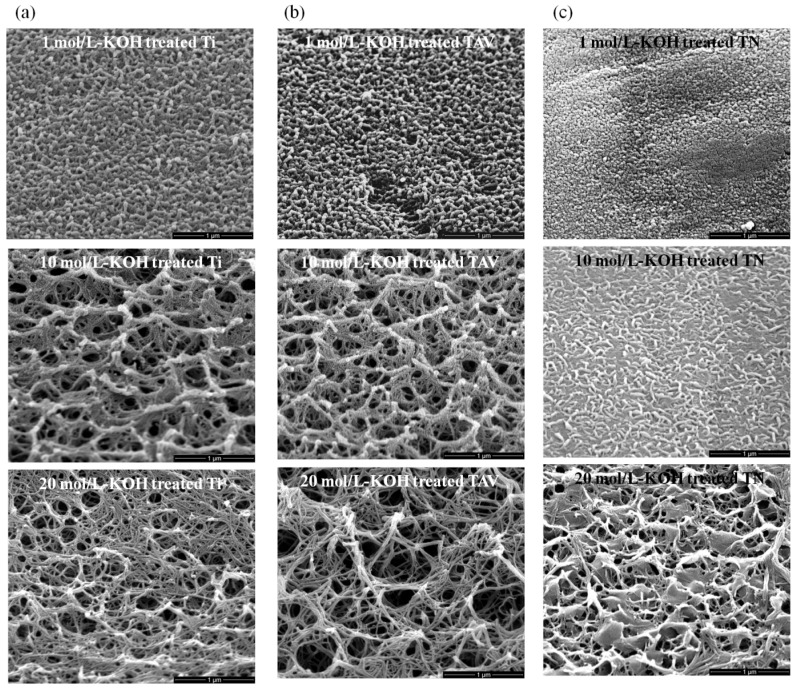
45° tilted scanning electron microscope (SEM) images of synthesized nanostructure. (**a**) SEM images of the Ti series after KOH treatment; (**b**) SEM images of the TAV series after KOH treatment; (**c**) SEM images of the TN series after KOH treatment. Ti and TAV metals which consist of more than 90% of Ti yielded elongated nanostructures of K-incorporated nTOFs (diameter: about 10 to 100 nm, length: several tens of micrometers) using 10–20 mol/L-KOH solution. In contrast, TN metal with 50% Ti content needed a KOH treatment with >20 mol/L-KOH solution. In short, 10 mol/L-KOH-treated Ti & TAV and 20 mol/L-KOH-treated TN showed similar morphology.

Figure 1 shows the scanning electron microscope (SEM) images of the surfaces of the Ti, Ti–Al–V (TAV) and TiNi (TN) substrates treated with 1, 10 and 20 mol/L-KOH solution at room temperature for 24 h. In our previous works, we have already described the morphology obtained from pure Ti plates after the KOH treatment of various concentrations [21,36,37]. Identical to these previous reports, 3D network structures consisting of elongated nanowires were formed on the surface of Ti plates (Figure 1a). The resulting network structures were strongly determined by the precise concentration of the KOH solution. Only within the window of 10–20 mol/L-KOH treatment could 3D network structures of nanowires be obtained. The diameter of these nanowires ranged from 10 to 100 nm, and their length was several tens of micrometers. Interestingly, using TAV substrates, very comparable morphology evolution was obtained at the same KOH conditions (Figure 1b). In contrast, the morphology obtained after treatment of TN substrates indicated that the condition for establishing nanowire structures was different from that of Ti and TAV samples. For the TN samples, the process window to successfully synthesize nanostructures was shifted up to higher KOH concentration ranges. After treatment with 1 mol/L-KOH solution, ball-like structures were formed on the TN substrates, while at the same conditions, thick and short nanowires were produced on the Ti, and TAV surfaces. Such thick and short nanowires could only be obtained using TN substrates when the KOH concentration was increased to 10 mol/L. The comparable 3D network structures with long and thin nanowires obtained from Ti and TAV substrates by treating with KOH with a concentration of <10 mol/L-KOH could be produced by treating TN substrates with 20 mol/L-KOH solution. Although the concentrations of the KOH solutions to produce nanostructures differ for the used initial materials, the trend of resulting morphology of nanostructures was almost the same: Short and thick nanowires formed first at a lower concentration, whereas at a higher concentration (>10 mol/L-KOH solution), 3D network structures with thin and long nanowires were favored, which disappeared at much higher concentrations. It has to be emphasized that our results show for the first time that various morphologies can be synthesized using various Ti-based materials via WCP by fine-tuning the treating KOH solutions.

**Figure 2:**
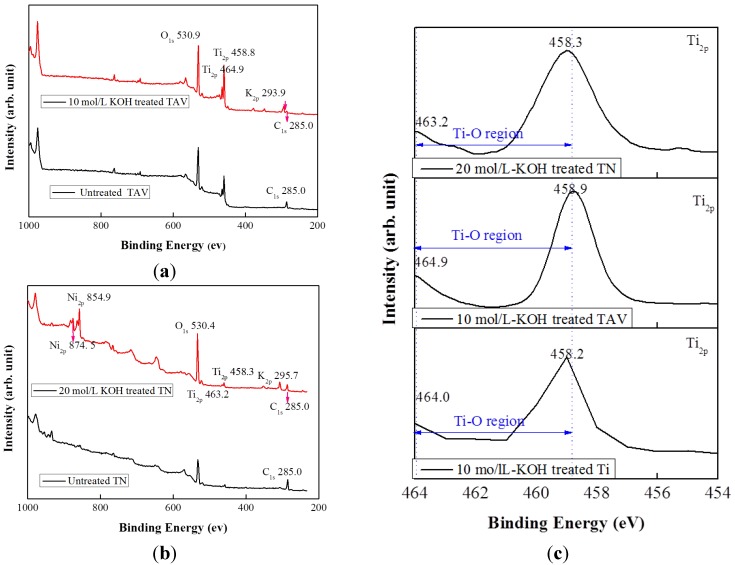

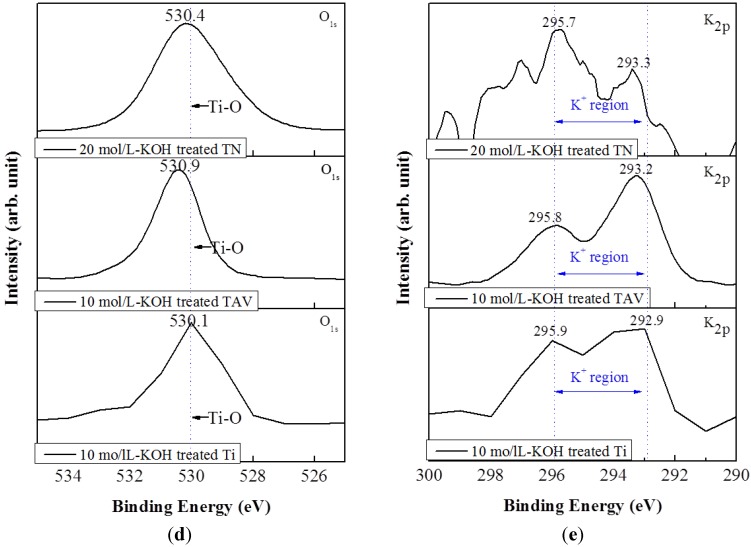
X-ray photoelectron spectroscopy (XPS) spectra of the surface of the Ti, TAV, and TN substrate within the same morphology. (**a**) Wide scan XPS spectra of the surface of the TAV substrate after 10 mol/L-KOH treatment; (**b**) wide scan XPS spectra of the surface of the TN substrate after 20 mol/L-KOH treatment; (**c**) Ti_2*p*_ spectra of the surface of the Ti, TAV and TN substrate; (**d**) O_1*s*_ spectra of the surface of the Ti, TAV and TN substrate; (**e**) K_2*p*_ spectra of the surface of the Ti, TAV and TN substrate. Compared with untreated substrates, K_2*p*_ peaks were clearly observed in all the KOH-treated Ti, TAV and TN substrates. This indicates that K-incorporated end products are formed by Ti–O–K bonding due to the chemical changing.

As already mentioned in the previous reports, K become incorporated into the resulting titanium oxide nanostructures due to the KOH treatment [23,38,39]. Thus, chemical bonds rearrange during the fabrication process and change the surface chemical compositions. In order to verify the surface chemical composition of the obtained nanostructured oxide films, X-ray photoelectron spectroscopy (XPS) analysis was performed. As already reported for Ti substrates [23], the incorporation of K into the titanium oxide films could be evidenced by the existence of the K_2*p*_ peak in the XPS spectrum. The XPS data obtained from the Ti substrate were in agreement with the previous results and are therefore not shown here. Figure 2a shows a wide range XPS spectrum of the surface of the TAV substrate subjected to the 10 mol/L-KOH solution. The XPS spectrum of the untreated TAV substrate is included for comparison. The untreated TAV substrate showed the Ti_2*p*_ doublet peaks of Ti_2*p*1/2_ and Ti_2*p*3/2_ at 453.8 eV and 458.9 eV, respectively, attributed to the Ti–Ti bond. However, the 10 mol/L-KOH-treated substrates showed these peaks at a higher binding energy of 458.7 eV and 464.4 eV due to the Ti–O bond formation. Note that an Al holder and an X-ray source of 600 W excitation were used to obtain the XPS data, and we slightly controlled the aperture. Because of this, the Al intensity was very low and detection was very difficult. The V_2*p*_ peaks could be detected at 509.1 eV on the untreated TAV substrate, which disappeared after the KOH treatment, indicating the removal of the alloying species Al and V during the KOH treatment and the formation of potassium titanates on the surface without incorporation of Al and V [45]. Conversely, the K_2*p*_ peak could clearly be detected at 293.9 eV in contrast to the untreated substrate. This peak position, however, does not match the pure K phase but rather the oxidized state K^+^, which can be explained by the formation of the Ti–O–K bonding [23]. The O_1*s*_ peak was detected on the untreated substrate surface due to the natural passive oxide layer, but a very strong O_1*s*_ peak appeared at 530.9 eV after the KOH treatment. This peak corresponds to the binding energy of the O_1*s*_ peak ascribed to the Ti–O bond, owing to the potassium titanates formed on its surface [46,47].

Figure 2b shows the wide range XPS spectrum of the TN substrates after treatment with 20 mol/L-KOH solution as well as the spectrum of the untreated substrate for comparison. The untreated TN substrate also showed the Ti_2*p*_ doublet peaks of Ti_2*p*1/2_ and Ti_2*p*3/2_ at 454.2 eV and 458.1 eV, respectively, which were very comparable with the TAV substrate. These peaks are attributed to the Ti–Ti bond. The 20 mol/L-KOH-treated substrates also showed a chemical shift towards higher binding energies, namely at 458.3 eV and 463.2 eV, respectively, again indicating the formation of Ti–O bonds in potassium titanates. Strongly pronounced Ni_2*p*_ doublet peaks of Ni_2*p*1/2_ and Ni_2*p*3/2_ were detected in the 850–890 eV region of the spectrum of the KOH-treated substrate, compared with the untreated substrate. Similar to the chemical shift to higher binding energy, as observed for the Ti_2*p*_ peak in the KOH-treated TAV substrate, the main Ni_2*p*_ peak also shifted to a higher binding energy of 854.9 and 874.5 eV due to the Ni–O bond formed, leading to the oxidation state of Ni^2+^ and Ni^3+^ [48,49,50,51]. This means that Ni oxide components coexist in the obtained oxide film, unlike the TAV substrates where the alloying species of Al and V are released during the KOH treatment. Hence, although TAV and TN are both Ti-based alloys, the behavior of its alloying species after the KOH treatment is different: most of Al and V are released, whereas a small amount may remain in the potassium titanate nanostructures. This is in agreement with the trend reported in literature, where up to 50% of Al and V release was observed during the KOH treatment [45]. In the case of TN substrates, although Ni is released from the alloy during the KOH treatment, the remnants of Ni exist in the obtained oxide films due to the large amount of Ni present in the TN substrate. This phenomenon can clearly be seen in the XPS spectrum by the occurring peaks of Ti_2*p*_ and Ni_2*p*_ at 458.3 eV and 854.9 eV in the KOH-treated TN substrates. These Ti_2*p*_ and Ni_2*p*_ peaks thus correspond to Ti^Ni–Ti^ and Ni^Ni–Ti^. This result is also consistent with the X-ray fluorescence (XRF) results, which reveal that there is no significant change in the content of Ni before and after the KOH treatment (data not shown). Comparably to the TAV-series, the K_2*p*_ peak is clearly absent in the untreated substrate, but could be detected at 295.7 eV after the KOH treatment. This result also clearly confirms the formation of the Ti–O–K bonding on the surface. The evolution of the O_1*s*_ peak is similar to that observed in TAV substrates: a very strong O_1*s*_ peak at 530.4 eV clearly demonstrates the strongly pronounced formation of Ti–O bonds owing to the conversion to potassium titanates after the KOH treatment. For pure Ti substrates, the synthetic process of potassium titanate nanostructures via the WCP was explained in the previous work [21]. To summarize briefly, Ti metal is partially dissolved by KOH solution and then forms Ti–O bonds. The involved chemical reactions are described as follows:

(1) TiO_2_ + OH^−^ = HTiO_3_^−^
(2) Ti + 3OH^−^ = Ti(OH)_3_^+^ + 4e^−^
(3) Ti(OH)_3_^+^ + e^−^ = TiO_2_·H_2_O +1/2H_2_
(4) Ti(OH)_3_^+^ + OH^−^ = Ti(OH)_4_
(5) TiO_2_·*n*H_2_O + OH^−^ = HTiO_3_^−^·*n*H_2_O

Since this process forms negatively charged HTiO_3_^−^·*n*H_2_O which attracts positively charged K^+^ ions, parts of the Ti–O bonds turn into Ti–O–K due to incorporation of K ions.

**Figure 3:**
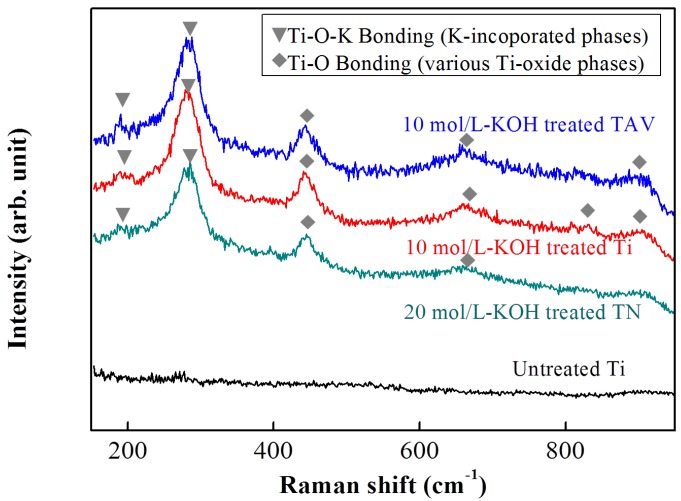
Raman spectra of the surfaces of 10 mol/L-KOH-treated Ti, 10 mol/L-KOH-treated TAV and 20 mol/L-KOH-treated TN and untreated substrates. Compared with untreated substrates, Ti–O–K bonds were clearly observed in all KOH-treated Ti, TAV and TN substrates. The general trend for structural changes is remarkably similar within the same morphology, indicating that the TiO_2_ phase, which is made of Ti–O bonds, and K-incorporated Ti–Oxide phase, made of Ti–O–K bonds, are generated by rearranging the atoms in the Ti metal. Note that the Ti–O–K bonds peaks were detected in the region <400 cm^−1^ and the Ti–O bonds peaks were detected in the region 400–1000 cm^−1^.

In order to study the generated chemical bond in more detail, a Raman analysis was performed. The Raman spectra of the KOH-treated Ti-, TAV- and TN-series are presented in Figure 3. For comparison, untreated Ti was used as reference and 10 mol/L-KOH-treated Ti & TAV and 20 mol/L-KOH-treated TN substrates were selected in order to correlate the same nanostructure morphology with the Raman signals. As can be seen in Figure 3, the gradual change of Raman spectra are remarkably similar for the samples with comparable morphology originating from different substrates and different WCP conditions, when compared with the untreated sample. In the present studies, a Raman spectrometer with the back-scattering configuration was used. Generally, Raman shifts are affected by the vibration of the electronic polarization of the constituents in the films, which depend on bonding characteristics such as the atomic distance and the bonding angle [52]. Hence, Raman results presented in Figure 3 suggest that the chemical changes occurring during the WCP synthesis process are the same for all starting materials, in agreement with the XPS results shown in Figure 2. Together with SEM and XPS results (Figure 1 and Figure 2, respectively), we can conclude that the nanostructures, formed from different starting metal substrates and under different WCP conditions, are not only of the same morphology but also contain the same chemical bonds. As we previously reported, nanostructures originating from Ti plates via the WCP consisted of two components, namely TiO_2_ phase containing Ti–O bonds and K- incorporated Ti–O–K bonds. Both were generated by rearrangement of atoms in the Ti metal during the WCP [23]. The K-incorporated Ti–O–K bonds give rise to a new peak in the Raman spectrum close to 280 cm^−1^, which is present in the Raman spectra of all samples (Figure 3). Consequently, the presence of the Ti–O–K bonds in all nanostructures can be concluded. Depending on the initial materials, this peak slightly shifts (Ti-series: 280 cm^−1^, TAV-series: 284 cm^−1^, TN-series: 273 cm^−1^). Besides, Raman peaks of various TiO_2_ phases could be found at higher values, namely for Ti series (442, 657, 815, and 917 cm^−1^), TAV series (446, 666, and 920 cm^−1^), and TN series (442 and 722 cm^−1^) [53,54,55]. The peaks at about 442 and 446 cm^−1^ were assigned to the Ti–O bending vibration involving three-fold oxygen coordination, whereas the peaks at about 657, 666, 722, 815 cm^−1^ were assigned to the Ti–O bending and stretching vibration involving two-fold oxygen coordination. The peaks at about 917, 920 cm^−1^ were correlated with the Ti–O stretching vibration involving non-binding oxygen, some of which were coordinated with K ions. The peaks of K-incorporated Ti–O–K bonds below 400 cm^−1^ and the peak at 445 cm^−1^, which are ascribed to the Ti–O bending vibration involving three-fold oxygen, gradually increased with increasing concentrations of KOH solutions [53,56,57,58]. The formation of the Ti–O–K bonds involves a rearrangement of the Ti–O bonds where K atoms are inserted into the space between Ti–O bonds. Ti–O–K bonds are formed with some of the broken Ti–O bonds whereas other Ti–O bonds are maintained. Hence, these measurements clearly evidence the formation of the (Ti–O–K) structure beside the TiO_2_ phases [52], in all Ti-containing substrates.

**Figure 4:**
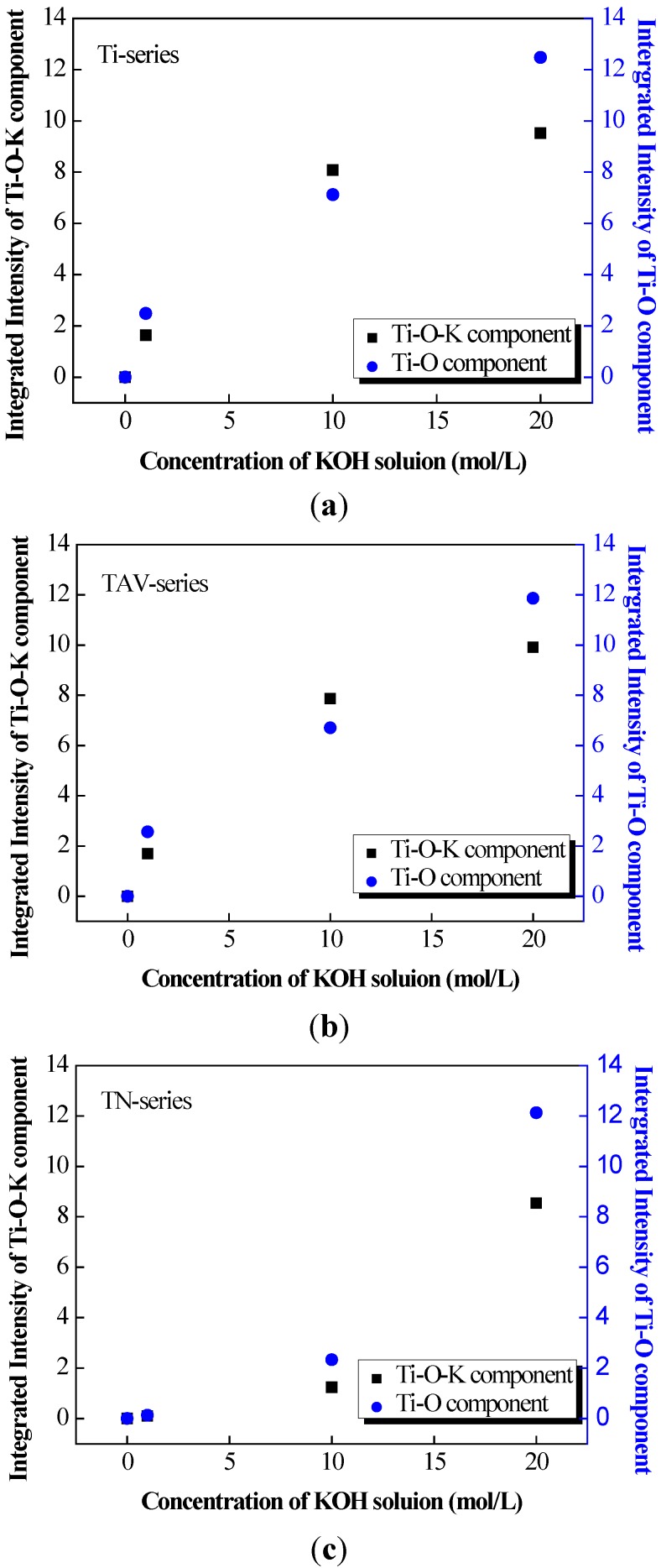
The integrated intensity of the Ti–O–K components and the Ti–O components in KOH-treated (1, 10 and 20 mol/L-KOH solution) Ti, TAV, and TN substrates estimated from the ambient Raman spectra. (**a**) The chart of the untreated and KOH-treated Ti substrates; (**b**) The chart of the untreated and KOH-treated TAV substrates; (**c**) The chart of the untreated and KOH-treated TN substrates. We can conclude that the amount of incorporated potassium is directly determined by the volume of the Ti–O–K component: the Ti–O–K component increased simultaneously with the increase in the concentration of the KOH solution.

Based on the results of the XPS and Raman data, we can conclude that the K-incorporated nTOFs could be obtained from the TAV and TN substrates via the WCP as from the pure Ti-substrates, following the same chemical reactions mentioned above. A rough estimate of the volume of the Ti–O components and Ti–O–K components in the oxide films produced after the WCP of Ti, TAV and TN samples can be obtained by integration of the Raman peaks. Figure 4 summarizes the evolution of the Raman peak intensities as a function of the KOH concentrations for the different samples. To be precise, for this estimate the relative intensities of the Ti–O–K contributions (Ti series: 280 cm^−1^, TAV series: 284 cm^−1^, TN series: 173, and 273 cm^−1^) and that of the various Ti–O phases (Ti series: 442, 657, 815, and 917 cm^−1^, TAV series: 446, 666, 827, and 920 cm^−1^, TN series: 722 cm^−1^) have been taken into account for both components, respectively [53,54,55]. As can be seen, the intensities clearly increase with higher concentration of the KOH solution for all different substrate materials. This phenomenon can be explained with the increase of the Ti–O and Ti–O–K components in the resulting oxide films together with the rearrangement of the chemical bonds after the WCP. For both Ti–O and Ti–O–K components, the integrated intensity follows the same trend and increases with the KOH concentration, indicating that the potassium titanates and TiO_2_ phase are synthesized simultaneously during the WCP and the amount of incorporated potassium is directly correlated with the amount of the Ti–O–K component. This result is in agreement with our previous observation on pure Ti metal plates, where Ti partly dissolved in alkaline solution, leading to synthesis of TiO_2_ substances. However, the new results illustrate that the general trend for volume changes of the Ti–O and Ti–O–K components is remarkably similar for different starting materials with various Ti content within the process window, leading to nanostructures of the same morphology. This result indicates that the various Ti-containing starting materials follow the same chemical reaction of WCP and that the Ti content in the initial metal determines the amount of Ti–O–K component formed.

**Figure 5:**
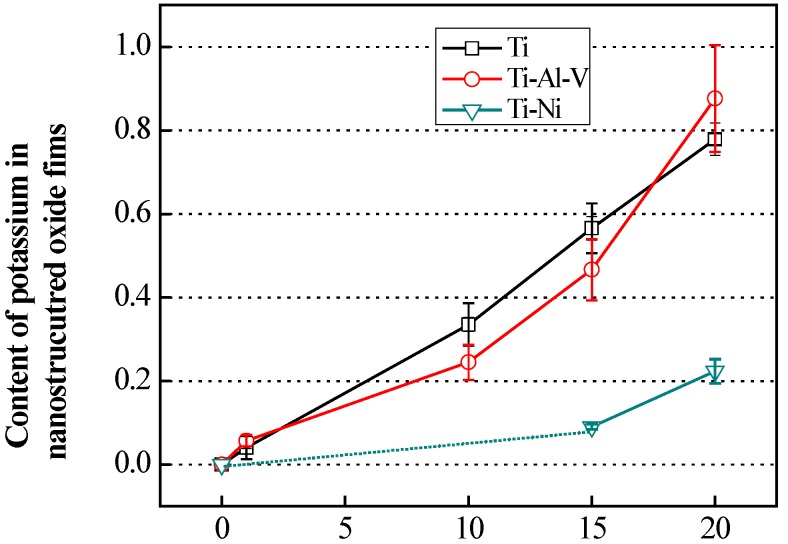
Content of potassium in obtained nanostructured oxide films from X-ray fluorescence (XRF). The general trend for content of potassium in nanostructures is remarkably similar with Ti and TAV series. However, the overall tendency of the K content in the TN series is shifted toward higher KOH concentration regions. The value of 10 mol/L-KOH-treated TN was below the limit of the measurement and therefore not visible, and cannot be seen in the figure. The shortage of Ti content can be compensated for by using higher concentrations of KOH solution, which promotes nanowires formation.

Figure 5 shows the results of the XRF measurements for the Ti-, TAV-, and TN- samples as a function of the K content. It is noteworthy that the evolution of the K content in the Ti and TAV series is almost the same, while again the TN series show a different trend. As already mentioned, the KOH-treated Ti and TAV series behave similarly at the same KOH concentration conditions, leading to very comparable morphology and structure of nanostructures. The fact that the evolution of the K content in the Ti and TAV series is also similar within the same KOH condition indicates that the Ti and TAV samples can be considered as very similar with respect to the WCP process. This observation also supports the Raman and XPS results showing Al and V mostly released from the alloy during the KOH treatment and leaving Ti behind to undergo the chemical reactions mentioned above. In contrast, the TN substrates behave differently. The morphology and structural properties of 20 mol/L-KOH-treated TN substrates were close to the results obtained from the 10 mol/L-KOH-treated Ti and TAV substrates. Hence, the chemical process window shifted to a higher concentration region. The XRF results clearly demonstrate that this shift also goes together with the K content: In the 20 mol/L-KOH-treated TN substrates, the K content is close to the value obtained from the 10 mol/L-KOH-treated Ti and TAV substrates. Taking these results into account, we can conclude that (i) the resulting morphology is strongly correlated with the K content in the nTOFs; and (ii) the K content is an essential parameter for the formation of 3D network structures with long and thin nanowires in the Ti, TAV, and TN samples.

The WCP reaction commonly occurs between the initial material and the solution, but the process window for producing 3D network structures greatly depends on materials and the process condition. This implies that there is a limitation in the reaction. The Ti content in the initial material in each series can be considered the key factor in fabricating the nanostructured morphology via WCP. Therefore, the similar Ti content in Ti and TAV substrates leads to very similar results for the WCP, unlike the TN substrates with significantly lower Ti content. In order to form titanium oxide, Ti has to dissolve first in alkaline solution to establish Ti–O bonds, which represent the main bonds of the nanostructures. It was reported that the structure of the potassium titanates consist of layered sheets containing Ti–O bonds linked to K atoms [59]. Consequently, we expect K to be partly incorporated into the oxide structures and to form the final structure on the surface of the treated substrates. Hence, the resulting morphology is governed by the amount of Ti–O bonds formed during the WCP. Indeed, TEM studies confirmed nanowires containing a layered structure (Figure S1). In agreement with the XRF data, electron energy loss spectroscopy and energy dispersive X-ray analysis revealed the presence of K in individual nanowires. Comparing the Ti and TAV series with the TN series, the TN substrates can only make a significantly smaller amount of Ti–O bonds because of the reduced Ti content. This shortage of Ti content can be compensated for by using higher concentrations of KOH solution, which strongly promotes Ti–O bond formation. Consequently, the 3D network structures with nanowires form at the higher KOH concentration. Thus, the amount of Ti in the Ti-based metals and incorporated K are the key factors in producing nanostructured morphology.

**Figure 6:**
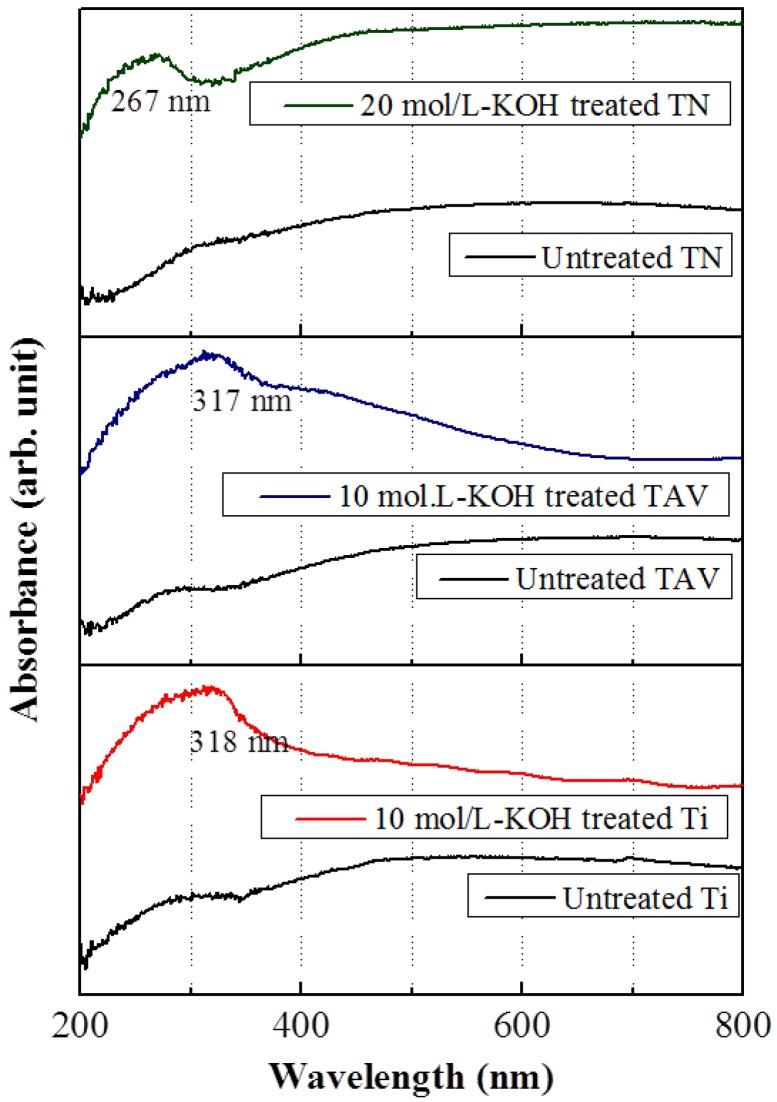
Absorption spectra of the untreated and KOH-treated Ti, TAV and TN substrates. In order to the electronic structure of the obtained nanostructured Ti, TAV and TN series within the same morphology, UV-vis measurements were carried out. A blue shift in the absorption peak was observed after the KOH-treatment in all series due to the quantum size effects in nanostructures. The incorporated K atom induced the rearrangement of the energy levels of the Ti–O components during the formation of the Ti–O–K components.

To investigate the electronic structure of the obtained nanostructured Ti, TAV and TN series, UV-vis measurements were carried out. Figure 6 presents UV-vis absorption spectra of the Ti, TAV, and TN series with the same morphology. The enhanced absorption peaks were clearly seen after the KOH treatment in all series, which indicate that a change of the optical band gap occurred after the KOH treatment of the substrates. From the absorption spectra, this change can be estimated following the direct correlation between the optical band gap energy *E* and the measured wavelength λ [60]:

*E* = *h*·*c*/λ

where the parameters *h* and *c* correspond to the Planck constant and the velocity of light, respectively. In the Ti series, the absorption peak was observed at approximately 360 nm corresponding to a band gap of 3.44 eV in the untreated substrates, while the absorption peak shifted to approximately 317 nm in the 10 mol/L-KOH-treated substrates. This corresponds to a modified band gap of 3.91 eV. In the TAV series, the absorption peak was observed at approximately 348 nm, which is consistent with a band gap of 3.56 eV in the untreated substrates. In the 10 mol/L-KOH-treated substrates, the absorption peak was observed at approximately 318 nm, corresponding to a band gap of 3.89 eV. In contrast, the absorption peak of untreated and 20 mol/L-KOH-treated TN substrates appeared at approximately 356 nm and 267 nm, corresponding to a band gap of 3.48 eV and 4.64 eV, respectively. Compared to the optical band gap reported in literature for TiO_2_ (3.20 eV) [61,62,63], it has to be emphasized that a blue shift of the absorption peak was observed in all series after the KOH-treatment. It is well known that a shift of the absorption peak depends on the atomic composition of metallic films and is related to the quantum size effects in nanomaterials [64,65,66]. Since the atomic rearrangement results in the nanostructures with Ti–O and Ti–O–K components during the WCP process, we think that the energy levels change due to the atomic rearrangement leading to a shift in the absorption peak. Consequently, the role of K atoms in the WCP can be considered as follows: K atoms are incorporated into the Ti–O bonds, thereby forming the Ti–O–K bonds. This reaction not only leads to nanostructures, it also induces a rearrangement of the energy levels of the Ti–O components, which influence the band gap of the Ti–O component. It is likely that K atoms do not directly contribute to the change of the band gap. Duan *et al.* demonstrated in their first-principles study of KNbO_3_ that the K electron states were located in the upper part of the conduction band (>8 eV) and in the lower part of the valence band (<−10 eV) [67]. Thus, the bottom of the conduction band and the top of the valence band were determined by Nb_4d_ and O_2*p*_ states and not influenced by K. Consequently, we can conclude that the blue shift observed in the vicinity of the TiO_2_ phase (around 3.2 eV) is determined by Ti and O states. More detailed study is needed in order to fully understand the origin of this blue shift. Nevertheless, this variation of the absorption property of the obtained nanostructured Ti-based oxide films is of great promise for tuning the electronic and optical properties for a wide range of future technological applications.

**Figure 7:**
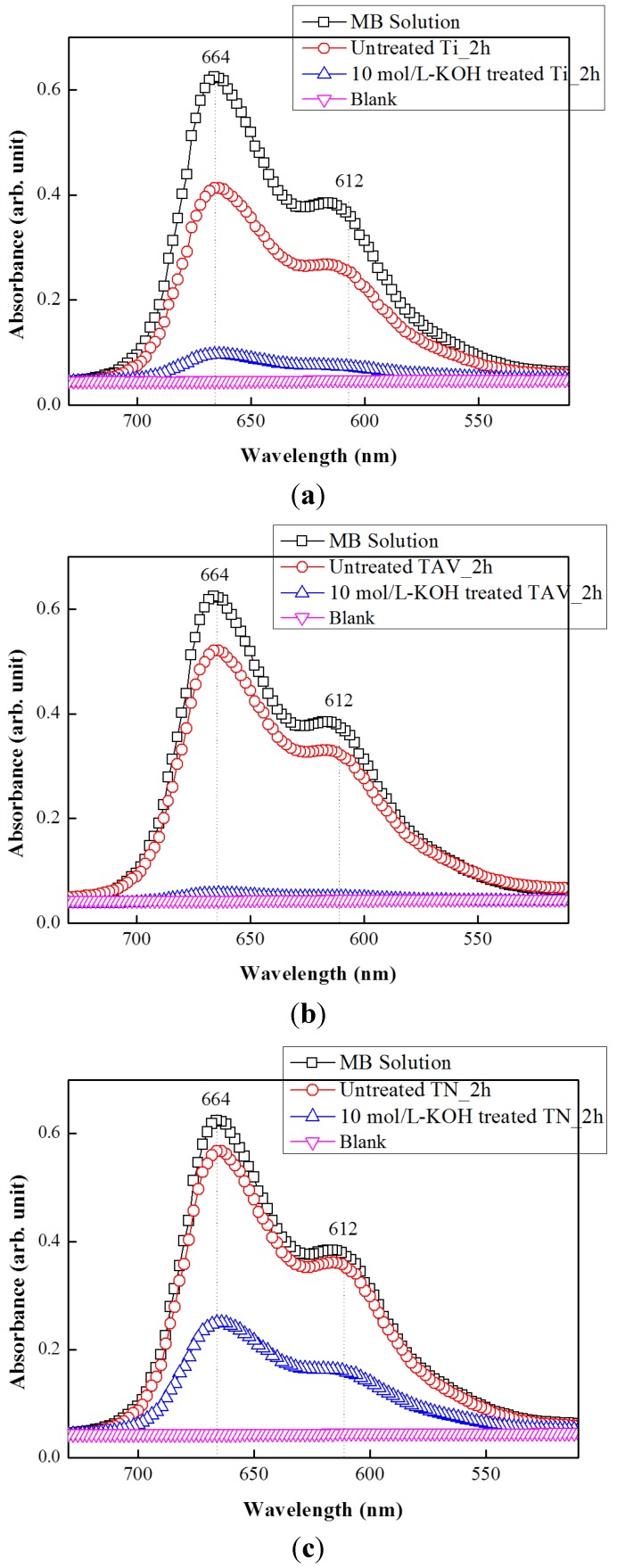
UV-vis absorption spectra of MB solution in the presence of the K-incorporated nTOFs and untreated sample. The intensity of the main absorption peak at 664 and 612 nm reflects the MB. The peak decreased after 2 h of degradation as a blank level, indicating the efficiency of a photocatalyst.

In order to examine the photocatalytic activity of the K-incorporated nanostructures obtained from the Ti, TAV and TN series, photodegradation of methylene blue (MB) was evaluated under UV light irradiation. The UV-vis absorption spectra presented in Figure 7 demonstrate the decomposition of MB dye leading to decolorization. For comparison, the absorption spectra of pristine MB aqueous solution as well as that of an untreated sample are also presented. The major absorption peaks appear at about 612 and 664 nm which are characteristic for MB dye [68]. Their decrease in intensity can be used as a direct indication for photocatalytically activated dye degradation. Indeed, all nanostructures produced from Ti, TAV, and TN alloys show a higher photocatalytic activity than untreated samples. This observation is in agreement with the remarkable photocatalytic properties reported for nanostructured titanium oxide materials with enhanced surface area and increased electron transfer ability [69,70]. In Figure 7 the results of 10 mol/L-KOH-treated Ti and TAV samples are presented together with that of the 20 mol/L-KOH-treated TN sample. As presented in Figure 1, all three samples yield comparable nanostructures under the respective WCP conditions. Interestingly, the 10 mol/L-KOH-treated Ti and TAV samples show a higher photocatalytic activity than the 20 mol/L-KOH-treated TN sample despite their similar morphology. The intensity of the MB peaks decreased to a blank level for Ti and TAV samples whereas it was reduced to about half for the TN sample after 2 h of UV exposure. This difference in Ti (or TAV) samples and TN samples can be explained as follows: Generally, the photocatalytic process is based on the photogeneration of electron-hole pairs, which will initiate redox reactions with the species adsorbed on the surface of the catalysts. In the photocatalytic process, OH radicals originating from the oxidation of OH^−^ or H_2_O by the photogenerated electron-hole pairs in the presence of oxygen have been considered as the major reactant responsible for the photocatalytic oxidation of organic materials and degradation of pollutants [71,72,73]. Consequently, improved electronic properties can be linked to enhanced photocatalytic activity. In our work, the electronic properties are governed by the amount of Ti–O–K components. Therefore, the 20 mol/L-KOH-treated TN sample, which has fewer Ti–O–K components, shows less pronounced photocatalytic activity. From these results, we can conclude that an efficient charge/energy transfer occurs in the KOH-treated samples under photo-irradiation and leads to the improved photocatalytic activity, as evidenced by the drastic diminishment of the UV absorption peaks. These results clearly demonstrate that the K-incorporated nTOFs can be a cost-effective, highly efficient, and environmental-friendly photocatalyst.

## 3. Experimental Section

### 3.1. Preparation of Nanostructured Titanium Oxide Films with Ti, Ti–Al–V Alloy, and TiNi Alloy

Commercially available, pure Ti metal, Ti–Al–V alloy, and TiNi alloy substrates were used in the present study. Although the substrates used in this work were all titanium-based metals, the total Ti content was different. Ti and Ti–Al–V plates were purchased from ChemPUR with the composition as mentioned in Table 1. TiNi was a hot-rolled plate with Ti:Ni ratio close to 50:50 but with some traces of Cu. Ti and TAV contained 100% and 90% Ti, respectively, whereas TN consisted of 50% Ti.

The detailed compositions are listed in Table 1. All the metal substrates, 10 × 10 × 1.0 mm^3^ in size, were polished with #400–#2000 SiC papers and washed with pure acetone and distilled water in an ultrasonic cleaner. Then, alkali treatment was performed by soaking all these substrates in 5 mL of KOH aqueous solutions with concentrations of 1, 5, 10, 15, 20, 25 and 30 mol/L at room temperature for 24 h. In this paper, we have mainly shown the results of the 1, 10 and 20 mol/L treated samples because they were most representative. For instance, the 10 mol/L-KOH-treated Ti and TAV samples and the 20 mol/L-KOH-treated TN samples showed significantly different morphology compared to 5 mol/L, which yielded rather similar morphology to the 1 mol/L treatment. After the alkali treatment, all the metal substrates were gently washed with distilled water.

**Table 1:** Chemical composition of the starting materials used in the present study.

Metal	Composition (wt %)				
	Ti	Al	V	Ni	Cu
Ti	Bal.				
Ti–Al–V	Bal.	6.45	4.16		
TiNi	Bal.			50	5

### 3.2. Characterizations

Changes in the surface structure, shape, and size of the Ti, Ti–Al–V, and TiNi specimens were observed by using a scanning electron microscope-focused ion beam (Dual Beam FEI NOVA SEM: FEI Company, ModelNoba 600 NanoLAb, Hillsboro, OR, USA) which were operated at 15 kV with top view and 45° tilted samples. To investigate the structural property of the obtained product, Raman spectroscopy (Renishaw, in Via Raman microscope) was performed using 514.5 nm Ar laser radiation as the excitation source. For analyzing the elements in all fabricated specimens, X-ray fluorescence (XRF: Philips, Model XRF Spectrometer Philips PW 2400, Amsterdam, The Netherlands) measurements were carried out using a powerful X-ray source (50 kV, 40 mA). Elemental composition and chemical state of the elements present in all specimens were determined by X-ray photoelectron spectroscopy (XPS: JEOL, Model JPS-9010 MC Photoelectron Spectrometer, Tokyo, Japan) with a focused monochromatic Al K αX-ray source (600 W) for excitation. The selected region spectra were recorded covering the Ti_2*p*_, O_1*s*_, K_2*p*_, Ni_2*p*_ and C_1*s*_ photoelectron peaks. The acquisition conditions for such high-resolution spectra were 20 eV pass energy with the step of 0.1 eV. In order to evaluate the optical properties and the electronic state of the obtained nanostructured Ti-based oxide films, UV-vis Spectroscopy (UV: JASCO Inc., Model JASCO V-570, Tokyo, Japan) was carried out. The optical density was measured in absorption condition.

### 3.3. Photocatalytic Activity Testing of the K-Incorporated nTOFs

Methylene blue (MB) at the concentration of 10 mg·L^−1^ was used as the testing solution. For the photocatalytic assessments, a UV exposure unit (LV202-E, Farnell, Leeds, UK) was used with a working area of the light source of 159 mm × 229 mm and the distance between the light source and the sample of about 10 cm. The UV output was 2 times 8 W with wavelength in the range 350–450 nm. To be precise, 350 µL of the MB solution was added onto the top of surface of the untreated Ti and K-incorporated nTOFs. The UV radiation was applied to the reaction sample plates for 2 h and the photocatalytic activities were evaluated by collecting the UV-vis (UV: Tecan Model infinite M2000 PRO, Männedorf, Switzerland) absorption spectra of the solution.

## 4. Conclusions

We fabricated nanostructured K-incorporated titanium oxide structures via WCP using the Ti, TAV, and TN substrates. Systematically the relation between the Ti content of the initial metal and the condition of WCP was studied, with the conclusion that these parameters strongly control the morphology and physical properties of the generated nanostructures. One-dimensional nanostructures could be obtained within a specific window of the process condition: Ti and TAV metals which contain more than 90% Ti yielded elongated nanostructures of K-incorporated titanium oxide films after treatment with 10–20 mol/L-KOH solution. In contrast, TN metal with about 50% of Ti content required a KOH treatment with >20 mol/L-KOH solution indicating that the process window shifted to higher concentration. For all samples the morphology evolution with the WCP condition was similar, but the precise Ti content of the initial material determined the amount of incorporated potassium as well as the KOH concentration at which elongated nanostructures were produced. This phenomenon can be explained as follows: a TiO_2_ phase consisting of Ti–O bonds and K-incorporated titanium oxide phase consisting of Ti–O–K bonds are generated after treatment with KOH solution. During this process, atoms in the Ti metal rearrange, forming Ti–O and Ti–O–K bonds. Furthermore, as demonstrated, the physical properties of the resulting material varied with the WCP conditions. In particular, the blue-shift of the UV-vis spectra indicated a quantum confinement effect as well as an increase of the optical bandgap. The K-incorporated nTOFs also exhibited high photocatalytic activity which can particularly be attributed to the enhanced charge/energy transfer owing to the present Ti–O–K components. These results demonstrate that the WCP is a simple and highly controllable technique for the fabrication of K-incorporated titanium oxide nanostructures. The possibility to precisely tune the morphology as well as physical properties by controlling the KOH concentration and by the selection of the starting Ti-based material makes this technique highly versatile and interesting, especially for diverse large-scale applications. Especially for the production of nanostructures to be used in photocatalysis, water treatment and potentially in biomedical applications, such as antibacterial control systems, the WCP holds great promise.

## Supplementary Materials

Supplementary materials can be accessed at: http://www.mdpi.com/2079-4991/5/3/1397/s1.
