# Supplementary material for: Preparation and Photocatalytic Activity of Potassium-Incorporated Titanium Oxide Nanostructures Produced by the Wet Corrosion Process Using Various Titanium Alloys
Source: Nanomaterials (Basel). 2015 Aug 21;5(3):1397–417. doi: 10.3390/nano5031397 (PMC5304627; doi:10.3390/nano5031397)
Supplement: Supplementary file 1 [file nanomaterials-05-01397-s001.pdf]

Supplementary Information

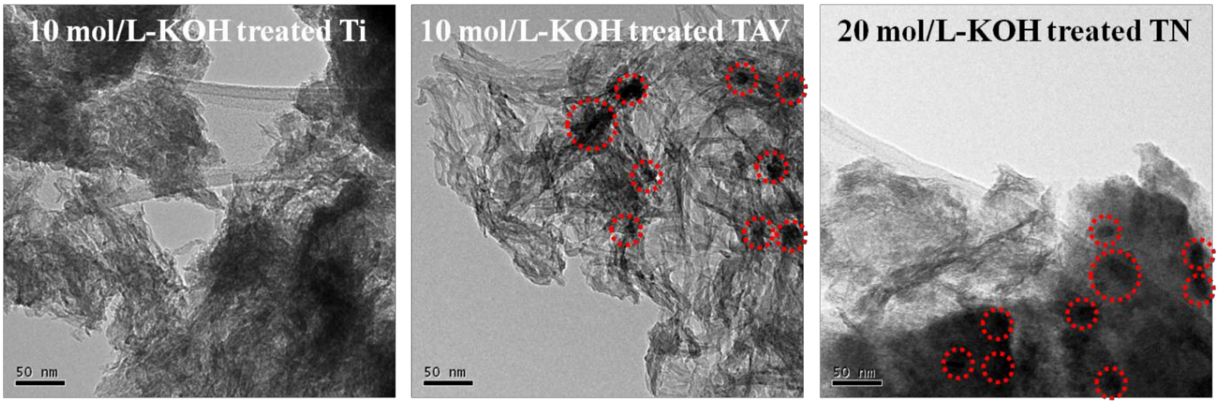

(**a**)

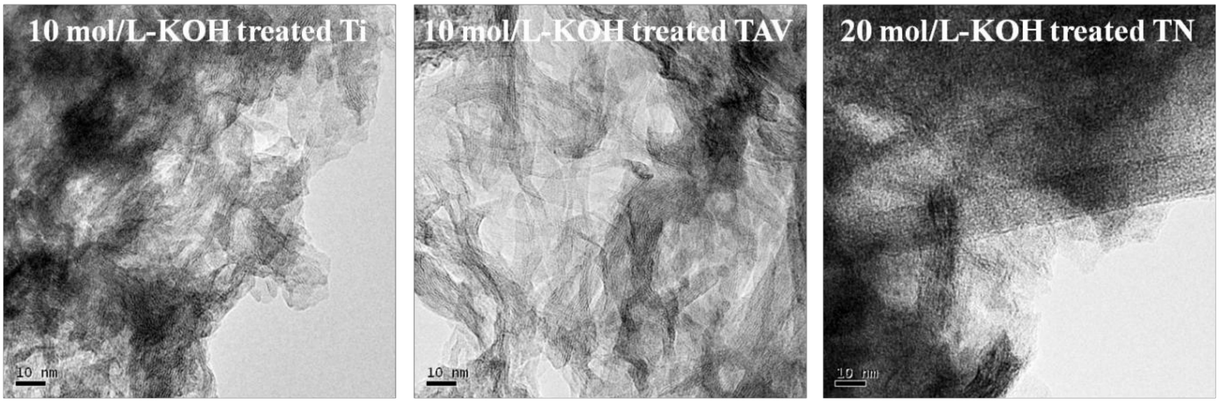

(**b**)

Figure S1. (**a**), (**b**) TEM images of microstructures of 10 mol/L-KOH treated Ti & TAV and 20 mol/L-KOH teated TN samples. TEM image show the similar layer structure of the nanowires within same morphology.

Microanalysis was analyzed by a transmission electron microscope (TEM: Philips, Model CM 200-FEG) at 200 kV of the accelerated voltage of electron. Figure a shows the dark field TEM images of microstructure of the 10 mol/L-KOH treated Ti & TAV and 20 mol/L-KOH treated TN. Within the same morphology, the similar network structures made of elongated nanofibers were observed in these samples. This phenomenon correspond with the morphology change studied by SEM. The alloying species were observed in the 10 mol/L-KOH treated TAV and 20 mol/L-KOH treated TN samples, compared with the 10 mol/L-KOH treated Ti sample. The round mark on the TEM images indicates the alloying species. It is noteworthy that the obtained endproducts include the alloying species, resulting an open possibilities for various applications with the properties of alloying species. The atomic structures of the nanofiber is further investigated using HRTEM (Figure S1b). HRTEM images show that the obtained products are basically layer structure with a layer spacing of 1.09 nm, respectively. It is reported that the structure of K-incorporated titanium oxide consist of layerstructures, which consist of Ti–O bonding layers linked by sharing edges and corners. K^+ ions are located between the layers. Therefore, the layer structures are formed by connecting corners of the Ti–O bonding layers to link the layers together [1,2].

Reference

1. Anderson, S.; Wadsley, A.D. The Crystal Structure of $K_2Ti_2O_5$. *Acta Chem. Scand.* **1961**, *15*, 663.
2. Ángel, A.G.M. *Advances in Ceramics—Electric and Magnetic Ceramics, Bioceramics, Ceramics and Environment*; InTech: Rijeka, Croatia, 2011; pp. 487–510.

© 2015 by the author; licensee MDPI, Basel, Switzerland. This article is an open access article distributed under the terms and conditions of the Creative Commons Attribution license (http://creativecommons.org/licenses/by/4.0/).
